# Overcoming Mechanical Blindness: Adaptive Surgical Instrument Design for the Heterogeneous Landscape of Uterine Fibroids

**DOI:** 10.34133/research.1385

**Published:** 2026-08-20

**Authors:** Jixuan Liu, Rong Liu, Xiaorong Xie, Ling Zhang, Yijun Zhao, Baiyang Sun, Yi Zhang, Shuwen Chen, Wenbin Chen, Yukun Liu, Siyi Jiang, Cuidi Chen, Chaoyang Sun, Jiajie Guo, Caihua Xiong

**Affiliations:** ^1^ Institute of Medical Equipment Science and Engineering, Huazhong University of Science and Technology, Wuhan, China.; ^2^ School of Mechanical Science and Engineering, Huazhong University of Science and Technology, Wuhan, China.; ^3^ Department of Obstetrics and Gynecology, Tongji Hospital, Tongji Medical College, Huazhong University of Science and Technology, Wuhan, China.; ^4^ School of Architecture and Urban Planning, Huazhong University of Science and Technology, Wuhan, China.; ^5^ School of Public Administration, Huazhong University of Science and Technology, Wuhan, China.

## Abstract

Uterine fibroids pose a major global health burden, affecting approximately 120 million women worldwide. While minimally invasive surgeries, such as electromechanical morcellation, preserve fertility, they are severely hampered by the risk of unpredictable tissue dissemination—a hazard that prompted a U.S. Food and Drug Administration black-box warning. Resolving this clinical dilemma requires a paradigm shift in instrument design. This review identifies a persistent “mechanical blind spot”: Current surgical tools operate with static parameters that fundamentally clash with the profound multi-scale mechanical heterogeneity of fibroids. Driven by FIGO-defined microenvironments and degeneration-driven remodeling, this extreme variability in tissue stiffness shapes the dynamic—and often hazardous—cutting challenges during surgery. To bridge this critical gap, we review the mechanobiological origins of this heterogeneity and analyze its direct impact on morcellation-induced fragmentation. We then synthesize multiscale mechanical quantification methods (ex vivo and in vivo) and evaluate the evolution of instrument-optimization strategies. Moving beyond conventional structural refinements, we highlight the potential of bio-inspired end-effectors for safe tissue interaction. Crucially, we propose a technological roadmap integrating these physical mechanisms with artificial intelligence—enabling preoperative “mechanical mapping” and real-time adaptive control. Taken together, this work offers new perspectives for the clinical management of this prevalent condition and provides a comprehensive theoretical framework for developing next-generation, mechanics-aware surgical ecosystems.

## Introduction

Uterine fibroids represent a pervasive and growing global health burden, affecting an estimated 120 million women worldwide with a lifetime risk of 70% to 80% [1]. The socioeconomic toll of this disease is profound. In the United States, annual costs surged to an estimated $42.2 billion in 2022, surpassing expenditures on breast and colon cancers [2]. Beyond its economic impact, the epidemiology of uterine fibroids is deeply complex, characterized by profound demographic disparities and rapidly shifting regional trends. Demographically, the disease burden is highly unequal. For instance, African American women experience faster tumor growth and undergo hysterectomies at twice the rate of their white counterparts [3]. Geographically, the disease footprint is expanding at an alarming rate, particularly in populous nations. In China, the disease is surging with an annual incidence rate of 0.46% (outpacing the global 0.27%), surpassing 1 million new cases in 2019 alone [4]. This confluence of high prevalence, rising incidence, substantial economic cost, and systemic health disparities underscores the urgent need for innovative and accessible solutions to this pressing public health challenge.

Currently, minimally invasive surgery (MIS) represents the preferred approach for managing uterine fibroids, with advantages of minimizing trauma and preserving fertility. However, this ideal approach is compromised by its core instrument, the electric morcellator, which is highly prone to scattering tissue fragments [5]. When these scattered fragments contain undetected malignant cells (e.g., sarcomas), this iatrogenic seeding drops patient 5-year survival rates from 73% to 21% [6]. Driven by a pivotal JAMA study reporting an almost 8-fold increase (odds ratio = 7.89) in the risk of sarcoma spread [7], the U.S. Food and Drug Administration issued a black-box warning in 2014, strongly cautioning against the routine use of morcellators [8]. This regulatory warning has driven the market withdrawal of leading morcellators without viable replacements [9]. Nevertheless, morcellation remains clinically indispensable to avoid highly invasive open surgeries, particularly for extracting massive fibroids (>5 cm) [5]. In this situation, clinicians adopt contained in-bag morcellation as a risk-mitigation strategy. Unfortunately, in-bag morcellation introduces new challenges: It prolongs surgical time by up to 45% [10], carries a non-negligible bag rupture rate of 9.6% [11], and fails completely for massive tumors (>8 cm) [12]. Consequently, this lack of safe and reliable devices forced a clinical regression to highly invasive open laparotomies [13] or conservative “wait-and-see” approaches despite risks of symptom progression [14], ultimately triggering a profound technological stagnation that demands an urgent engineering breakthrough.

To achieve this breakthrough, we must confront the physical root of this iatrogenic hazard: a profound solid-mechanics failure at the tool–tissue interface. Specifically, it exposes the “mechanical blindness” of statically designed instruments, which we define as the systemic inability to perceive real-time tissue stiffness, feed cutting forces back as control signals, and adapt kinematic parameters intraoperatively. This functional deficiency goes far beyond the static physical mismatches inherent in basic tool–tissue interactions; it represents a systemic loss of sensing, feedback, and adaptive capabilities, which we term “mechanical blindness”. Uterine fibroids are not mechanically uniform entities. Depending on their pathological subtypes and degenerative states, they exhibit extreme spatial gradients in stiffness, viscoelasticity, and fracture toughness [15]. However, current morcellators operate with fixed kinematic parameters (e.g., constant blade speed) alongside rigid blade geometries. This “one-size-fits-all” cutting mechanism inevitably fails across variable mechanical landscapes. When forced through these extreme tissue gradients, this non-adaptive mechanism stalls or excessively tears the tissue, converting routine cutting forces into uncontrolled fragmentation. A glossary of key mechanical terms is provided at the end of the manuscript.

Overcoming this mechanical bottleneck requires a paradigm shift toward mechanics-informed, tissue-adaptive instrument design. As demonstrated by applications such as stent radial forces graded by plaque mechanics [16] and porous orthopedic implants optimized for bone stiffness [17], this paradigm is already reshaping the broader field of biomedical engineering. In this context, uterine fibroid morcellation serves as a compelling macro-to-micro physical testbed. Its pronounced tissue heterogeneity and complex device–tissue interactions provide an unparalleled scenario for translating biomechanical insights into next-generation, tissue-adaptive fibroid morcellators, ultimately paving the way for a biomechanics-driven intelligent surgery framework.

To bridge the critical gap between clinical pathology and advanced engineering, this review presents a targeted, biomechanics-driven blueprint for fibroid morcellation. We systematically analyze the origins and characteristics of biomechanical heterogeneity, its clinical consequences, and the specific demands it places on surgical tools. By synthesizing state-of-the-art multiscale mechanical quantification techniques, bio-inspired structural optimizations, and artificial intelligence (AI)-driven control algorithms, we chart a roadmap for next-generation uterine fibroid morcellators, transitioning toward biomechanically adaptive and predictive-integrated intelligent systems. Ultimately, this work provides a conceptual framework and outlines a forward-looking roadmap for the development of next-generation, mechanics-aware surgical systems that can better address the challenges of extreme soft-tissue heterogeneity.

## The Dual Origins of Mechanical Heterogeneity

### The spatial dimension: FIGO classification as a mechanical niche

**Fig. 1.**
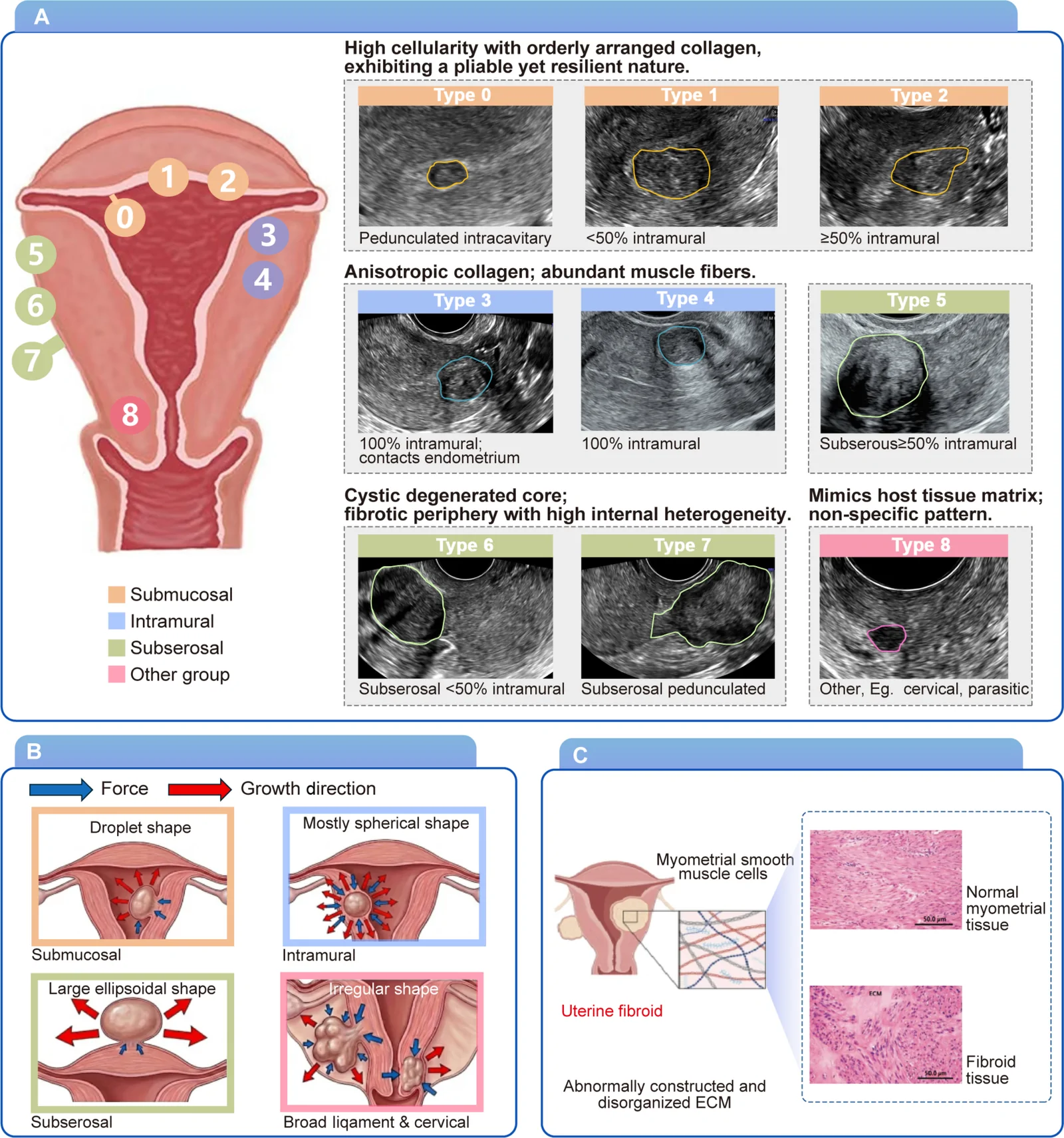
Classification and biomechanical characteristics of uterine fibroids. (A) Anatomical locations (FIGO types 0 to 8) and corresponding ultrasound imaging features. Submucosal (types 0 to 2, orange): Characterized by high cellularity and orderly arranged collagen, exhibiting a pliable yet resilient nature (type 0: pedunculated intracavitary; type 1: <50% intramural; type 2: ≥50% intramural). Intramural (types 3 and 4, purple): Feature anisotropic collagen with abundant muscle fibers (type 3: 100% intramural contacting endometrium; type 4: fully intramural). Subserosal (types 5 to 7, green): Often exhibit a cystic degenerated core and fibrotic periphery with high internal heterogeneity (type 5: ≥50% intramural; type 6: <50% intramural; type 7: pedunculated subserosal). Other (type 8, pink): Mimics host tissue matrix with nonspecific patterns (e.g., cervical or parasitic). The anatomical schematic is adapted from Bajaj et al. [83], and the ultrasound images are original data provided by the authors. (B) Schematic illustration of mechanical forces acting on fibroids according to their location, showing growth directions (red arrows) and opposing compressive/tensile forces (blue arrows). (C) Histological comparison between normal myometrial tissue (abundant, orderly arranged smooth muscle cells) and uterine fibroid tissue (abnormally unstructured and disorganized extracellular matrix), adapted from Kurt et al. [24] and Bulun [84].

The FIGO classification system provides a standardized anatomical descriptive framework for uterine fibroids [18,19]. As summarized in Fig. 1A, its primary clinical utility lies in guiding surgical strategy. However, beyond mere anatomical labeling, FIGO classification serves as a potential predictor of a fibroid’s biomechanical niche. The specific location within the uterus may influence the physical constraints and principal stress directions a fibroid experiences during growth (Fig. 1B), which is hypothesized to affect its macroscopic morphology and extracellular matrix (ECM) microstructure [20].

Submucosal fibroids (types 0 to 2) are confined by the rigid uterine cavity and subjected to high circumferential pressure. According to the official FIGO classification [18], type 0 submucosal fibroids are defined as intracavitary lesions attached to the endometrium by a narrow stalk (stalk diameter ≤10% of the mean diameter of the leiomyoma). This pedunculated morphology—a globular mass with a slender stalk—is distinctly different from the spherical, broad-based shape typically observed in subserosal fibroids, which is conceptually consistent with the distinct mechanical niches in which these fibroids develop.

As illustrated in Fig. 1A, the ultrasound images of different FIGO types—annotated with colored outlines—visually demonstrate these morphological differences. Submucosal fibroids (types 0 to 2, orange outlines) typically present as elongated, pedunculated masses within the uterine cavity, whereas subserosal fibroids (types 5 to 7, green outlines) generally exhibit spherical, broad-based shapes expanding into the abdominal cavity. This morphological contrast reflects the different mechanical environments in which these fibroids develop.

Intramural fibroids (types 3 and 4) grow under dynamic stress driven by myometrial contractions. It is hypothesized that this continuous anisotropic stimulation may contribute to a denser, more aligned ECM arrangement, which may in turn contribute to higher tissue stiffness. This hypothesis is conceptually consistent with the established observation of increased stiffness and altered ECM in fibroid tissues [20], and is further supported by studies showing that mechanical strain can dynamically realign collagen fibers [21] and that myometrial contractions impose location-dependent forces on fibroids [22].

Subserosal fibroids (types 5 to 7) expand with minimal constraint into the abdominal cavity, readily forming spherical masses. Their rapid volumetric growth frequently outpaces vascular supply, predisposing them to internal ischemia and a characteristically sparse, disorganized ECM architecture [23] (Fig. 1C).

This mechanical confinement is hypothesized to influence ECM organization, as supported by studies demonstrating that mechanical loading can modulate ECM crosslinking, tissue stiffness, and structural organization [24,25]. To preliminarily test this biomechanical hypothesis, we have initiated quantitative AFM mechanical characterization across different FIGO types. Our preliminary data indicate measurable differences in both background stiffness and localized extremes across these types, with the stiffness heterogeneity index (SHI = Max/Avg) revealing distinct mechanical profiles at different anatomical locations. The detailed experimental results, including representative nanomechanical mapping images and corresponding stiffness parameters, are summarized in Fig. S1 and Table S1. However, direct evidence linking specific FIGO types to distinct ECM structural phenotypes is currently lacking, and this remains an important area for future investigation.

Therefore, the FIGO classification offers a conceptual framework linking anatomical space with the local mechanical environment, providing a systematic view of the geometric constraints that may contribute to a fibroid’s mechanical niche [15]. In turn, this niche may govern location-specific stimuli that differentially orchestrate ECM remodeling, although this remains a hypothesis rather than a proven mechanism. This spatial framework also suggests a potential link between mechanical constraints and degeneration—such as ischemic changes in rapidly expanding subserosal types—which is consistent with the hypothesis that spatiotemporal constraints contribute to metabolic failure and pathological adaptation [23]. We note that direct evidence linking specific FIGO types to distinct ECM structural phenotypes is currently lacking, and this remains an important area for future investigation.

### The metabolic dimension: Ischemia-driven mechanical remodeling

**Fig. 2.**
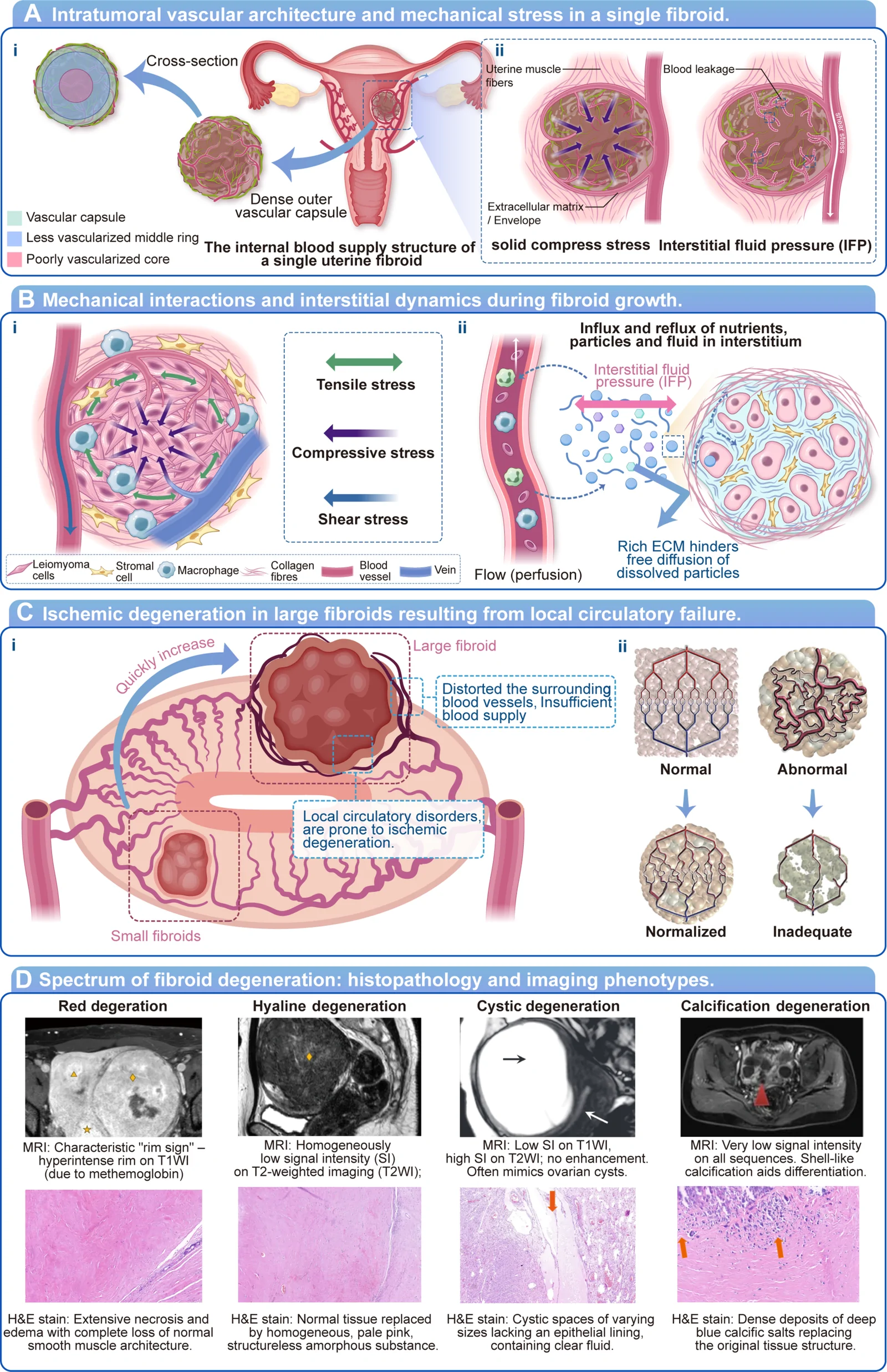
Biomechanical and vascular determinants of fibroid degeneration and their imaging correlates. (A) Intratumoral vascular architecture and mechanical stress. (i) Zonated blood supply: outer vascular capsule, hypovascular middle ring, and avascular core. (ii) Mechanical milieu defined by solid compressive stress and interstitial fluid pressure (IFP). Adapted from Slotman et al. [85] and Johnson et al. [86]. (B) Mechanical interactions and interstitial dynamics during fibroid growth. (i) The fibroid experiences and exerts complex mechanical stresses (shear, compression, and tension). (ii) Interstitial fluid pressure (IFP) drives nutrient convection, but diffusion is hindered in extracellular matrix (ECM)-rich regions, predisposing the tumor to ischemia. Adapted from Agrawal et al. [87]. (C) Ischemic degeneration in large fibroids resulting from local circulatory failure. (i) Rapid growth distorts local vasculature, causing core ischemia and subsequent degeneration. (ii) Normalized versus inadequate vascular perfusion, illustrating how aberrant blood supply dictates tissue viability. Adapted from Bulman et al. [88] and Jain et al. [89]. (D) Spectrum of degeneration. Corresponding histopathological [hematoxylin and eosin (H&E)] and radiological (MRI) phenotypes of 4 classic degeneration types: red, hyaline, cystic, and calcific. MRI images are adapted from Kaushik et al. [90], Wen et al. [91], and Slotman et al. [85]. H&E images are provided by the authors.

Fibroid degeneration represents a profound pathological failure of mechanical and metabolic homeostasis [26]. As illustrated in Fig. 2, the fibroid blood supply exhibits a 3-layered zonation, establishing a gradient of increasing solid compressive stress and interstitial fluid pressure (IFP) from the periphery to the core (Fig. 2A). During growth, vascular abnormalities and impaired lymphatic drainage collectively elevate IFP. Meanwhile, the dense ECM hinders pressure-driven diffusion, trapping the core in a state of chronic nutritional deprivation and metabolic waste accumulation (Fig. 2B). When fibroid expansion breaches a critical threshold, this prolonged mechano-metabolic imbalance precipitates hemodynamic decompensation, driving an ischemic cascade that culminates in core degeneration (Fig. 2C).

Crucially, this ischemic insult produces diverse pathological adaptations that radically rewrite the fibroid’s internal mechanical landscape (Fig. 2D). For instance, chronic ischemia predominantly leads to hyaline degeneration [26]^28^ [low T2 signal on magnetic resonance imaging (MRI)], causing uniform tissue stiffening (isotropic hardening) that fiercely resists cutting blades. In contrast, red degeneration [27] (characterized by “rim sign” on MRI) evolves from hemorrhagic infarction, while cystic [28] and myxoid [29] degenerations (displaying high T2 signals) arise from tissue liquefaction. Mechanically, these processes introduce highly localized compliance or fluid-filled voids, creating extreme internal structural gradients. Furthermore, extreme variants like calcification [30] and sarcomatous change [31] exhibit rock-like hardness or rapid structural disintegration. Without specific MRI markers, these mechanical extremes remain exceedingly difficult to differentiate preoperatively.

To address these diagnostic challenges, current research must bridge the gap between biomechanics and imaging phenotypes. For degenerations with established radiological signatures, quantifying the link between their mechanical basis and MRI manifestations improves diagnostic objectivity. Conversely, for imaging blind spots such as calcification and sarcomatous change, their extreme mechanical characteristics—such as peak tissue hardness or rapid structural disintegration—can serve as novel biophysical markers for detection. Ultimately, characterizing this mechanical heterogeneity overcomes the limitations of purely morphological assessments. It provides the specific physical parameters necessary to guide mechanics-informed surgical instrumentation and functional diagnostics.

It is worth emphasizing that beyond the anatomical constraints provided by FIGO location, the mechanical heterogeneity of fibroids is also heavily influenced by degeneration-related tissue remodeling and local vascular supply [24,25]. For instance, hyaline degeneration tends to induce isotropic hardening, while cystic or myxoid changes create highly compliant fluid-filled voids, and calcification introduces localized rock-like stiffness [23,30]. These distinct degeneration subtypes alter tissue consistency through changes in collagen cross-linking, hydration, and structural integrity.

## Quantitative Methods for Assessing Mechanical Properties of Uterine Fibroids

### Establishing the baseline: Ex vivo ground truth and preoperative mapping

**Fig. 3.**
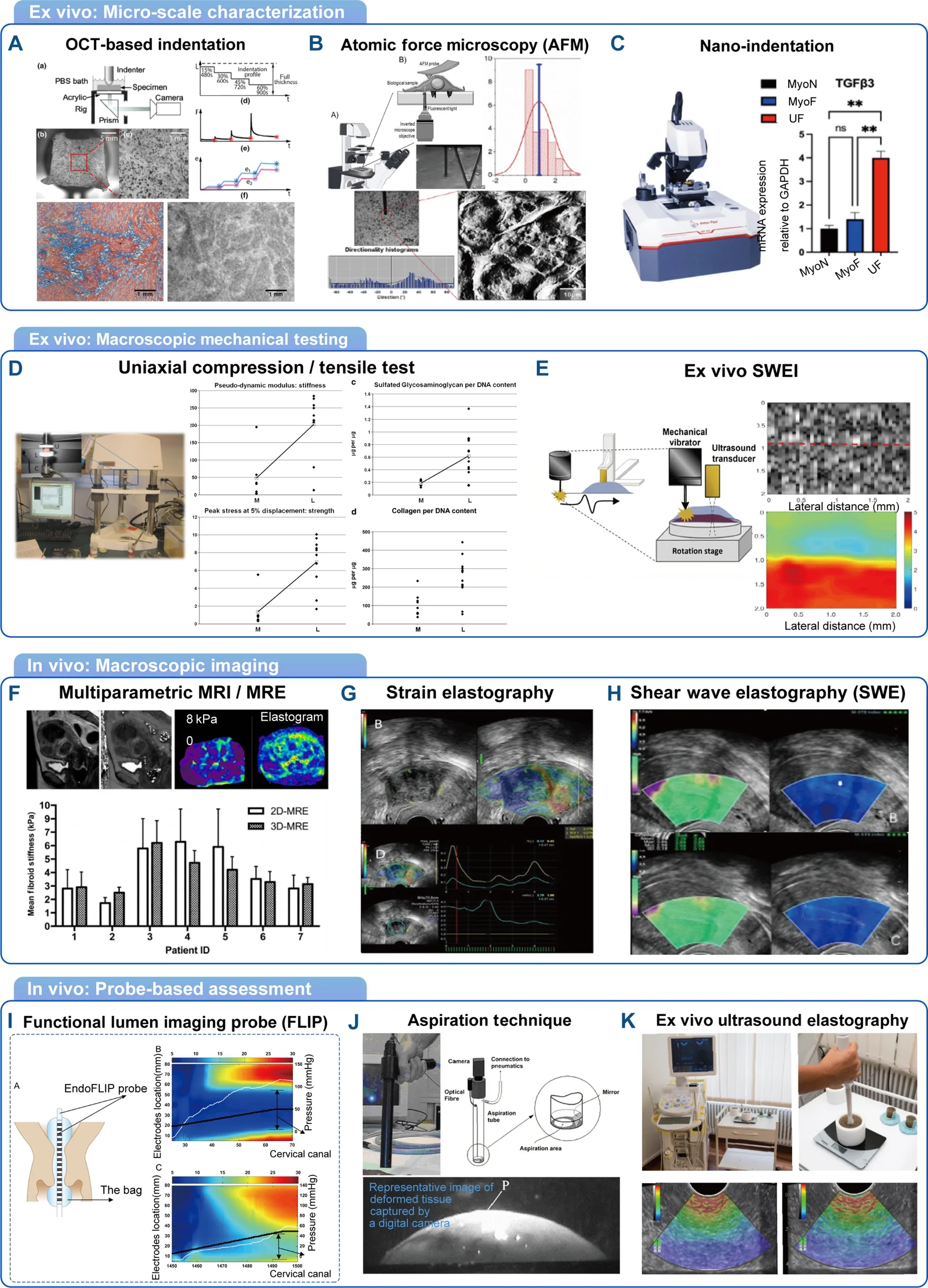
Multi-scale mechanical assessment techniques in gynecological tissue. (A) OCT-based indentation [35], (B) atomic force microscopy (AFM) [32], and (C) nano-indentation [34] for Ex vivo micro-scale characterization of cellular and regional stiffness variations. (D) Uniaxial compression/tensile testing [36] and (E) shear wave elastography imaging (SWEI) [92] for Ex vivo macro-scale mechanical testing of constitutive stress–strain parameters and macroscopic stiffness maps. (F) Multiparametric MRI/MRE [37], (G) strain elastography [38], and (H) shear wave elastography (SWE) [38] for In vivo macroscopic imaging to map spatial mechanical heterogeneity. (I) EndoFLIP system [41], (J) aspiration techniques [40], and (K) localized elastography [93] for In vivo probe-based assessment providing direct tissue–instrument mechanical feedback.

Accurately quantifying the mechanical heterogeneity of fibroids is fundamental to optimizing clinical management (Fig. 3). The biomechanical “ground truth” relies heavily on ex vivo characterization. Micro-scale techniques—such as atomic force microscopy (AFM) [32] and nano-indentation [33]—quantitatively resolve cellular-level stiffness variations and pathological softening [34]. Concurrently, macro-scale uniaxial testing [35] and shear wave elastography imaging (SWEI) characterize nonlinear biomechanical behaviors (e.g., >130% strain-stiffening) and extract hyperelastic constitutive parameters essential for engineering simulations [36].

Translating these mechanical baselines into clinical practice relies on noninvasive in vivo macroscopic imaging, functioning as a “virtual palpation” tool. Multiparametric MRI, magnetic resonance elastography [37] (MRE), and ultrasound elastography (USE) [38] map deep-seated stiffness heterogeneity, linking mechanical properties to tumor phenotypes and predicting treatment responses [e.g., high-intensity focused ultrasound (HIFU) ablation efficiency [39]]. However, while these macroscopic modalities provide excellent preoperative roadmaps, their physical constraints and limited spatial resolution preclude them from offering direct, real-time mechanical feedback during surgical tissue resection.

### The translational bridge: In vivo probe-based assessment

Bridging the critical gap between preoperative mechanical mapping and intraoperative surgical execution requires localized, probe-based assessment (Fig. 3D). These techniques provide real-time, site-specific mechanical feedback directly at the tissue–instrument interface.

Physical interaction-based methods deliver tangible mechanical measurements. For example, aspiration tests [40] and the functional lumen imaging probe (FLIP) [41] quantify dynamic surface stiffness and localized tissue distensibility under negative or positive pressure. Furthermore, emerging “smart” optical and microstructural sensors—including dynamic fluorescence [42], diffuse reflectance spectroscopy [43], and optical coherence tomography (OCT) [44]—enable near-histological microstructural evaluation to delineate collagen-rich boundaries and vascular anomalies in real time.

Crucially, the miniaturization and integration of these localized sensing technologies directly into surgical end-effectors represents a pivotal translational leap. By embedding mechanical and microstructural perception into cutting instruments, the clinical paradigm can shift from relying on passive preoperative maps to deploying context-aware, mechanically intelligent surgical tools capable of adaptive intraoperative decision-making.

## The Challenges Posed by Mechanical Heterogeneity to Surgical Efficacy

**Fig. 4.**
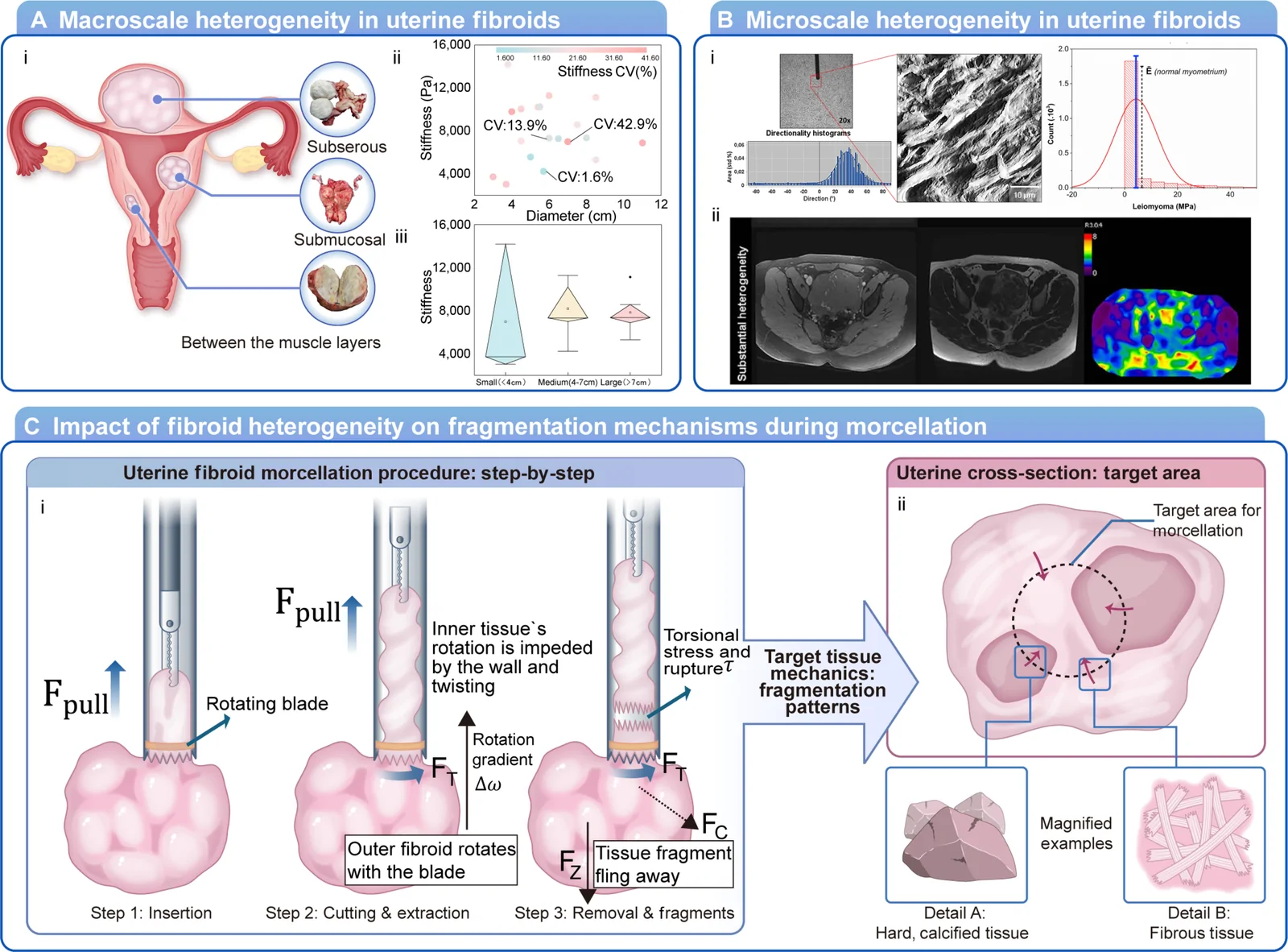
Multi-scale mechanical heterogeneity of uterine fibroids and its consequences during morcellation. (A) Macroscopic heterogeneity. (i) Anatomical locations and gross morphology. (ii and iii) Stiffness–diameter scatterplot and size-stratified box plots demonstrating extreme inter- and intra-tumoral stiffness variations (approximately 2 to 16 kPa). Redrawn from tabular data in Jayes et al. [45]. (B) Microscopic heterogeneity. (i) AFM mapping of the local nanomechanical landscape [32]. (ii) Ultrasound-based elasticity imaging mapping spatial variations in the macroscopic elastic modulus [37]. (C) Morcellation-induced fragmentation driven by mechanical mismatch. (i) Biomechanics of mechanical morcellation. As the rotating blade engages tissue under traction (Fpull), the impeded inner strand creates a rotation gradient (Δω) and torsional stress (τ). This accumulated stress, combined with the blade torque ($F_{t}$) and the axial feed force ($F_{z}$), ruptures the tissue and propels fragments via the resultant force ($F_{c}$). (ii) Resulting fragment morphologies dictated by target tissue mechanics: brittle, small fragments from hard/calcified tissue (detail A), versus larger, irregular strands from fibrous tissue (detail B).

Uterine fibroids exhibit an extremely broad range of macroscopic tissue stiffness (3,028 to 14,180 Pa) with substantial intra- and inter-tumoral variation [coefficient of variation (CV): 1.6% to 42.9%] [45], as detailed in Fig. 4A. This macroscopic variability fundamentally originates from the microscopic ECM landscape, which can be visualized via surface mapping techniques (Fig. 4B). Specifically, differences in collagen cross-linking, fiber alignment, and interstitial fluid mechanics collectively distort the typical soft-tissue stress–strain curve [46]. They combine to create a biphasic, viscoelastic mosaic of varying fracture toughness [47]. For example, regions of dense collagen are hyperelastic, while cell-rich areas remain highly compliant [48]. Under mechanical load, this profound structural heterogeneity makes the overall mechanical response highly unpredictable. Fractures propagate preferentially along the weak interfaces between these differing micro-domains, ultimately dictating an uncontrolled fragmentation pattern.

When fixed-parameter surgical instruments (e.g., constant speed and torque) engage this complex landscape, the cutting process is severely compromised. Because comminution efficiency correlates strongly with intrinsic fracture toughness [49], blade performance plummets in high-strength regions. This mechanical mismatch causes speed drops, stalling, or “skidding”, prompting excessive manual force [46]. Critically, forcing tissue to fracture along microstructural weak interfaces (e.g., cell-fiber boundaries) [50] generates distinct fragment morphologies. As Fig. 4C demonstrates, heterogeneous cutting resistance induces a “tissue rotation–fragment dispersion” cycle governed by nonlinear torque variations [51]. Clinically, local stiffness ultimately dictates this fragment morphology: High-stiffness areas undergo brittle fracture (producing sharp fragments), while low-stiffness areas yield to ductile tearing (creating flocculent micro-fragments).

Consequently, this multi-scale heterogeneity undermines surgical safety by directly disrupting the dynamic cutting process. The primary clinical hazard is the uncontrolled generation and dissemination of these fragments: Ductile tearing produces irregular micro-fragments with traumatically activated edges, exhibiting heightened viability that drastically elevates peritoneal seeding risks [52]. While this mechanical mismatch also compromises complete resection and boundary precision [45], the core failure lies in conventional instruments’ inability to safely navigate varying fracture toughness. Ultimately, this uncontrolled tissue–tool interaction establishes a clear imperative for developing adaptive, mechanics-aware surgical instrumentation.

## Impact of Mechanical Heterogeneity on Surgical Outcomes: Evidence from Clinical Retrospectives

Clinical retrospective analyses suggest a potential association between fibroid mechanical heterogeneity and surgical outcomes. Variations in tissue stiffness and composition may be associated with measurable differences in procedural outcomes, which could influence current interventions across 3 critical dimensions.

Compromised surgical efficiency: Mechanical heterogeneity may prolong key procedural steps and stresses instrument integrity. The resection of firm or voluminous fibroids requires markedly extended operative time across both surgical [53] and energy-based modalities [54]. Because static instrument parameters fail to adapt to dynamically variable tissue resistance, this mismatch may delay procedures and, in extreme cases, lead to instrument failure or compel the use of suboptimal, time-intensive techniques [55].

Undermined treatment completeness: Beyond impairing efficiency, heterogeneity may interfere with boundary delineation and energy deposition. In surgical enucleation, dense, fibrotic tissue obscures the fibroid–myometrium interface, which may increase the risk of residual disease and early recurrence [56]. In noninvasive energy-based therapies (e.g., focused ultrasound), variable acoustic and mechanical properties result in inconsistent ablation volumes—particularly in hyperintense or calcified subtypes—potentially leading to treatment failure [57].

Elevated complication risks: Mechanical heterogeneity may amplify clinical risks through 2 principal pathways: uncontrolled fragmentation and collateral damage. During mechanical morcellation, differences in tissue fragility may predispose to the generation of high-risk fragments and can breach containment systems, raising the potential for tissue dissemination [58]. Simultaneously, the higher energy doses or mechanical forces required to treat highly resistant tissue proportionally increase the risk of collateral injury, including thermal damage to adjacent structures and greater intraoperative blood loss [59].

In summary, the available clinical evidence provides preliminary indications that mechanical heterogeneity is a potential determinant of surgical outcomes, suggesting a link between tissue physics and procedural performance. These consistent limitations underscore the importance of developing mechanics-informed strategies to improve surgical safety and efficacy. A detailed synthesis of the supporting clinical studies is presented in Table 1. It should be noted that these findings are predominantly retrospective and warrant cautious interpretation.

**Table 1.** Impact of mechanical heterogeneity on surgical outcomes: Evidence from clinical retrospectives. The evidence summarized in this table is primarily derived from retrospective clinical observations and should be interpreted as indirect and preliminary.

Impact dimension	Manifestation of mechanical heterogeneity	Clinical findings/observations
Surgical efficiency	High hardness/toughness	Prolonged procedure time: Firm fibroids (>10 cm) increase morcellation time by 30–50% [53]. Large masses (≥300 g) extend laparoscopic surgery to ~162.5 min [94]. MRgFUS requires higher energy (up to 5,400 J) and prolonged duration (up to 6.8 h) [54].
	Calcification/extreme hardness	Instrument failure: Morcellator blade fracture during resection [94].
	Severe sclerosis/giant mass	Strategy alteration: Time-consuming “layered resection” to prevent instrument overload [55].
Treatment completeness	Blurred mechanical boundary (e.g., MED12 mutation)	Incomplete enucleation: Obscured tumor–myometrium interfaces elevated residual disease and recurrence risk [56].
	Extreme firmness	Misdiagnosis and poor planning: Preoperative misclassification risk, leading to suboptimal surgical modality selection [53,57].
	Acoustic/hydration heterogeneity (type 3, T2 hyperintense)	Ablation failure: High water content causes ultrasound scattering in MRgFUS (nonperfused volume ratio ~28.5%), risking volume rebound [55,95]. Calcified subtypes resist both FUS and UAE [96,97].
Complication risk	Low hardness/friability	Tissue dissemination: Morcellation generates minute particles (<2 mm), elevating intra-abdominal seeding risk (0.12–0.94%) for occult malignancies [58].
	High resistance/torque	Containment breach: Excessive cutting torque causes containment bag micro-perforations (up to 33% rate), facilitating malignant cell escape [12,98].
	High energy demand (FUS-related)	Collateral thermal injury: Requires ~2.1× higher energy, increasing skin burn risks [59,96]. Deep/adjacent masses correlate with nontarget thermal damage [54,95].
	Deep/paracervical location and giant mass	Adjacent organ injury and hemorrhage: Increases urinary (1.1%) and intestinal (0.2%) injury risks. Resection of large masses causes significant blood loss (average 405 ml) and high transfusion rates (up to 28%) [94,99].

FUS, focused ultrasound surgery; MRgFUS, magnetic resonance-guided focused ultrasound; UAE, uterine artery embolization

## Engineering Innovations for Adaptive Interventions

The severe clinical challenges posed by mechanical heterogeneity—namely, unpredictable tissue fracture and instrument stalling—necessitate a paradigm shift from static, “one-size-fits-all” tools to adaptive, mechanics-aware surgical systems. As a factual baseline for understanding the current engineering gap, we have summarized the technical parameters and static limitations of representative current morcellators in Table S2. The survey shows that the vast majority of currently available devices operate with fixed kinematic parameters and lack real-time tissue modulus sensing, which highlights a fundamental incompatibility with the extreme mechanical heterogeneity of uterine fibroids. Achieving this requires a multifaceted engineering strategy that integrates foundational cutting theory, topological structural design, and mechanism-driven bio-inspiration.

### Establishing the engineering foundation: Theoretical modeling and empirical validation

Precise physical quantification is the cornerstone of engineering optimization. Surgical cutting theory converts qualitative clinical observations into measurable parameters (Table 2). Foundational models transition concepts like “sharpness” into physical metrics [e.g., blade sharpness index (BSI)], predicting the precise relationship between tool geometry and required cutting force [60].

**Table 2.** Core framework and evolution of cutting theoretical models for surgical instruments

Model paradigm	Core formula/parameters	Target mechanism	Relevance to adaptive design
Orthogonal cutting (Gao et al. [100])	$F_{p}=\frac{G_{c}\cdot v^{0.34}}{\tan^{1.2}\alpha }F_{p}:$ Penetration force, α: Rake angle	Linear cutting with scalpels	Predicts cutting force based on blade velocity and rake angle; forms the physical baseline.
Blade sharpness index (McCarthy et al. [62])	$\mathrm{BSI}=\frac{r^{2}}{\delta_{c}}$ r: Blade tip radius, $\delta_{c}:$ Critical indentation depth	Tool–tissue interface mechanics	Optimizes blade tip radius to delay tool wear and reduce stalling in dense collagen.
Compression-cutting (Zhou and McMurray [60])	$F_{\mathrm{cut}}=k\cdot \sqrt{\frac{G_{c}Et^{3}}{r}}$ $G_{c}:$ Fracture energy, *t*: Tissue thickness, *k*: Sharpness coeff.	High-frequency rotary morcellation	Quantifies the critical role of tissue modulus *E* in driving cutting force; mathematically explains static stalling in hard fibroids.
Heterogeneous adaptation (Rogers et al. [20])	$F_{\mathrm{adj}}=\eta \cdot F_{\text{base}}$ $\left(\eta =\frac{E_{\text{tumor}}}{E_{\text{normal}}}\right)$	Tissue-specific force calibration	The ultimate basis for closed-loop adaptive control; uses modulus ratio (η) to dynamically scale cutting force.

These theoretical models are continuously refined through multimodal empirical data. High-speed digital image correlation (DIC) maps dynamic crack propagation, while force and electromyography (EMG) sensors quantify real-time tool–tissue interaction forces. By integrating theoretical heterogeneous adaptation models [20] (e.g., modulus ratio adjustments) with real-time empirical validation, researchers can establish the precise force-feedback benchmarks necessary for designing adaptive surgical instrument controls [61].

### Mechanical and kinematic optimization via finite element analysis

Building on these theoretical foundations, finite element analysis (FEA) offers a computational approach to re-engineer blade geometries and instrument kinematics for heterogeneous soft tissues. Structurally, parametric FEA has demonstrated that reducing the blade tip radius (e.g., from 20 to 12 μm) or optimizing the rake angle can lower initial cutting energy by up to 40%, shifting stress peaks away from delicate tissues [62].

Kinematically, topology optimization introduces variable cross-section designs and pseudo-rigid-body continuum joints [63]. These advancements expand the kinematic workspace and increase torque resistance (e.g., via internal toothed rings in morcellators) [51], effectively suppressing the dangerous “tissue rotation” cycles described previously.

### Bio-inspired mechanisms for dynamic tissue–tool interaction

**Fig. 5.**
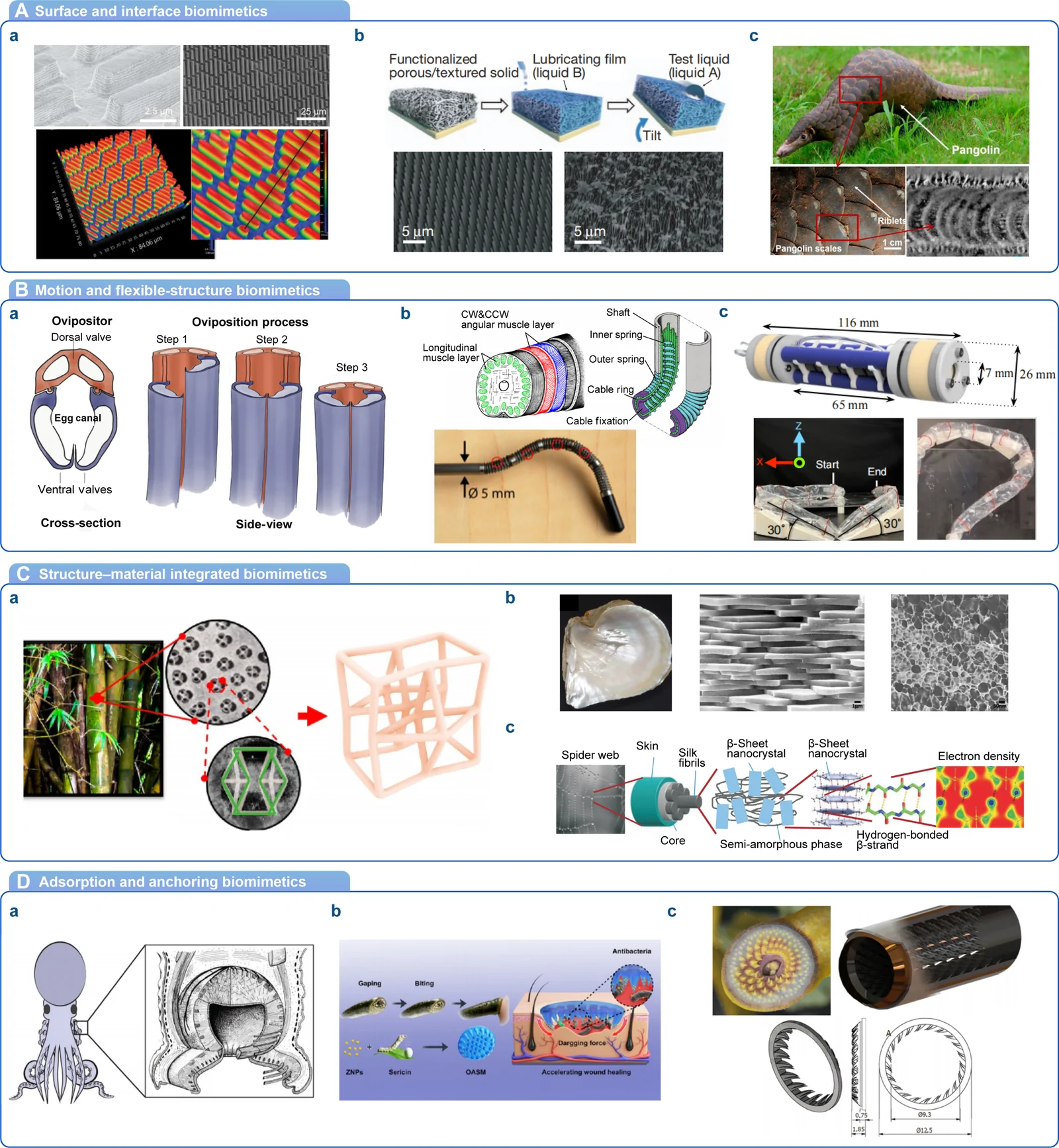
Biomimetic strategies addressing key mechanical challenges in surgical instrument design. (A) Surface and interface biomimetics for friction and adhesion control. (a) Shark-skin-inspired microstructures for drag reduction [64]. (b) Pitcher-plant-inspired SLIPS for anti-adhesion [65]. (c) Pangolin-scale-inspired microstructures for blade anti-adhesion [66]. (B) Motion and flexible-structure biomimetics for confined-space navigation. (a) Insect ovipositor kinematics [67]. (b) Squid-tentacle-inspired flexible architectures [68]. (c) Inchworm-inspired soft navigation mechanisms [69]. (C) Structure–material integrated biomimetics for torque and fatigue resistance. (a) Bamboo-inspired gradient porous structures [70]. (b) Nacre-inspired multi-scale layered structures [71]. (c) Spider-silk-inspired nanoconfined β-sheet crystals [72]. (D) Adsorption and anchoring biomimetics for tissue fixation and fragment containment. (a) Octopus-sucker-inspired soft suction gripper [73]. (b) Lamprey-teeth-inspired microneedles for anchoring [74]. (c) Passive gripping teeth integrated at the morcellator tip to constrain rotation [51].

The inherent conflict between static instrument operation and the dynamic mechanical environment of uterine fibroids manifests as 4 key clinical challenges: friction-induced blade stalling, torque overload, uncontrolled tissue rotation, and fragment scattering. Biological systems, whose structures embody solutions to similar mechanical problems, offer a source of design principles to address these challenges. As illustrated in Fig. 5, these strategies can be organized into 4 complementary domains, each offering a conceptual pathway to specific mechanical deficits.

Surface and interface biomimetics (addressing friction-induced blade stalling): This domain engineers the instrument–tissue interface to control friction and adhesion. Templates illustrated in Fig. 5A—shark-skin-inspired microstructures for drag reduction [64], pitcher-plant-inspired slippery liquid-infused porous surfaces (SLIPS) for low-friction anti-adhesion [65], and pangolin-scale-inspired microstructures for anti-adhesion [66]—demonstrate precision surface engineering. Their tribological principles could inform future blade modifications; however, their direct translation to morcellator applications remains unexplored.

Motion and flexible-structure biomimetics (addressing uncontrolled tissue rotation and confined-space navigation): Replicating biological movement improves instrument adaptability in confined spaces [e.g., the abdominal cavity, cranial base, or gastrointestinal (GI) tract]. Prototypes inspired by insect ovipositor kinematics [67], squid-tentacle-like architectures [68], and inchworm-inspired soft navigation [69] inform continuum manipulator design. While these offer conceptual pathways for navigating complex anatomical corridors—and could mitigate tissue rotation during cutting—their integration into rotary morcellation systems remains an open engineering question.

Structure–material integrated biomimetics (addressing torque overload and device fatigue): Mimicking the gradient architecture of bamboo [70], the “brick-and-mortar” structure of nacre [71], or the nanoconfined β-sheet crystals in spider silk [72] could enable composite metamaterials combining high rigidity with lateral compliance. These hierarchical designs demonstrate how controlling structure across multiple length scales enhances mechanical resilience. In the long term, such structures could improve the fatigue life of surgical instruments under highly variable mechanical loads.

Adsorption and anchoring biomimetics (addressing fragment scattering and tissue displacement): This domain develops strategies for temporary fixation on wet tissues without traumatic crushing. An octopus-inspired expandable soft suction gripper enables nondestructive grasping [73], while lamprey-teeth-inspired microneedles provide unidirectional anchoring [74]. Beyond these active strategies, passive mechanical constraints—such as a ring of gripping teeth integrated at the instrument tip—can also physically constrain the tissue mass when it begins to rotate [51], thereby mitigating uncontrolled tissue rotation.

Collectively, these biomimetic approaches serve a dual purpose: They provide a conceptual framework to rethink instrument–tissue interactions, and in a few cases—such as the passive gripping teeth directly translated from the lamprey oral disc—they offer concrete engineering blueprints that have already been prototyped and validated. However, direct translation into morcellator applications remains an open challenge, requiring further biomechanical modeling and device-level integration. The transition from biological inspiration to surgical hardware is complex, but it offers a promising pathway toward adaptive, mechanics-aware instruments. Figure S2 illustrates one such conceptual mapping using the lamprey as a representative paradigm; a deeper exploration of this bio-inspired adaptation is presented in Pillar 3.

## Future Perspectives: Overcoming “Mechanical Blindness”

The current safety and efficacy bottlenecks in minimally invasive fibroid surgery stem from a deeper limitation: the “mechanical blindness” of conventional instrument systems. Current tools cannot sense, interpret, or adapt in real time to the extreme mechanical heterogeneity of the target tissue. Therefore, the future breakthrough lies not in marginally optimizing static tools but in developing a new generation of “mechanically intelligent” adaptive surgical systems. This paradigm shift will be built upon 3 foundational pillars. An integrated overview of how these pillars interconnect is provided in Fig. S3.

Pillar 1: AI-enabled preoperative “mechanical mapping” and risk prediction

Future surgical planning must incorporate a “mechanical prognosis” beyond anatomical imaging. To realize this, a robust “mechanical-imaging” atlas must be built through large-scale paired studies, a crucial step given that AI applications in this domain remain nascent. This atlas would map precise ex vivo mechanical parameters—such as elastic modulus and viscoelastic properties—onto features from multi-sequence MRI and USE. Shear-wave elastography (SWE) has already demonstrated that fibroids significantly alter uterine stiffness in vivo [75]. Magnetic resonance elastography (MRE) offers a proven framework for noninvasive in vivo mechanical mapping [76]. At the microscopic level, emerging techniques like μElastography are now making precise ex vivo mechanical measurements more accessible [77]. Together, these tools—ranging from clinical ultrasound to advanced MRI and high-resolution ex vivo platforms—provide the data streams necessary to construct the atlas.

With this atlas in place, deep learning architectures [e.g., convolutional neural networks (CNNs) and Transformers] could integrate radiomic features, clinical parameters, and limited intraoperative sampling data to produce multimodal predictive models. Early attempts along this line have already shown promise, such as using multiparametric MRI to predict fibroid growth risk [78]. The eventual goal is for these algorithms to serve as intraoperative “safety sentinels”, alerting the surgical team to abrupt stiffness gradients that may trigger high-risk “skipping” fragmentation. These abrupt mechanical gradients are structurally consistent with real microscale ECM heterogeneities visualized by synchrotron radiation phase-contrast micro-computed tomography [79], and corroborated by ex vivo micromechanical testing [45]. For instance, a recent multidimensional model that integrated T2-weighted imaging (T2WI), diffusion-weighted imaging (DWI), and clinical data distinguished uterine sarcoma from benign fibroids with an area under the curve (AUC) of 0.96 [80]. Although this serves a diagnostic rather than a mechanical prediction purpose, it proves that the technical infrastructure for fusing multiparametric MRI, clinical variables, and AI is sufficiently mature to support future mechanical mapping [81]. Once mature, such integrated models could mark a pivotal shift from reactive complication management to proactive risk avoidance.

Pillar 2: Multimodal in situ sensing and real-time adaptive control

At the core of this mechanical intelligence is a closed-loop “perception–decision–execution” cycle. Integrating multimodal sensors at the instrument tip could enable real-time detection of tissue boundaries and mechanical properties. Diffuse reflectance spectroscopy has been validated for tissue boundary discrimination in upper GI cancer [43], and impedance probing has been used for real-time tissue differentiation in neurosurgery [44]. Both techniques could, in principle, be adapted to fibroid morcellation. Given constraints of size, sterility, and cost in direct tip integration, inferring tip forces from handle-mounted sensors via machine learning offers a practical alternative, as demonstrated in robotic uterine manipulation systems [82].

In the decision stage, sensory input can be coupled with compression-cutting fracture mechanics models [60] to trigger real-time adaptive control. For example, if cutting stress approaches a critical fracture threshold—e.g., when encountering calcified zones—the system could initiate a rapid deceleration protocol (sub-second to second-level, contingent on motor dynamics). Implementation would leverage advanced robust control algorithms from industrial robotics (e.g., adaptive sliding-mode or model predictive control). Translating these to the viscoelastic responses of fibroid tissues requires precise calibration. Meanwhile, advances in instrument kinematics and compliant mechanism design [63] could facilitate the hardware needed to physically execute these adaptive motions, although the closed-loop control architecture itself remains to be developed.

In the execution stage, it is hypothesized that such adaptive adjustments could suppress uncontrolled fragment dispersion and tissue spinning caused by torque accumulation [61] by modulating cutting kinematics before critical thresholds are reached. Nevertheless, direct experimental validation is required in future studies.

Pillar 3: Deep integration of bio-inspired end-effectors and smart materials

While AI provides the intelligent system’s “brain”, executing adaptive commands requires a fundamental leap in instrument hardware. Biological systems offer design principles for addressing core engineering challenges of fibroid morcellation, such as friction-induced blade stalling, torque overload, uncontrolled tissue rotation, and fragment scattering. Rather than listing biomimetic concepts exhaustively, we present the lamprey feeding apparatus to illustrate how biological mechanisms can be translated into engineering blueprints for adaptive morcellation.

As illustrated in Fig. S2, the lamprey’s tubular oral funnel closely mirrors the cylindrical shaft of a laparoscopic morcellator. Its keratinous dentition forms an annular rasping unit capable of feeding on host tissues with diverse mechanical properties (hard scales versus soft flesh), an operational scenario analogous to cutting heterogeneous fibroids. Crucially, the lamprey mouth creates a sealed negative-pressure compartment that simultaneously anchors the organism and prevents prey tissue fragments from escaping. This combination of graded mechanical cutting and active fragment containment provides a conceptual blueprint for a morcellator that could reduce friction-induced stalling while actively managing debris.

Beyond the lamprey paradigm, other biomimetic domains remain promising but currently conceptual. Surface engineering inspired by shark skin and related microstructured surfaces [64] may inform tribological modifications, while shape-memory alloys or electroactive polymers could enable adaptive stiffness modulation. Additionally, passive mechanical constraints, such as gripper teeth at the distal tip [51], demonstrate how simple structural modifications can mitigate uncontrolled tissue displacement.

The convergence of these 3 pillars—AI-driven preoperative mapping, real-time intraoperative adaptive control, and bio-inspired smart material design—defines a coherent roadmap toward genuinely intelligent, mechanics-aware surgical systems. This convergence not only charts a conceptual direction but also points to practical engineering entry points: curating paired mechanical-imaging datasets for AI training, miniaturizing and integrating validated sensor technologies into the morcellator tip, and initiating computer-aided design (CAD) modeling and benchtop prototyping by translating analogous biological blueprints into device design. While these pillars focus primarily on engineering solutions, a truly comprehensive future perspective also requires recognizing the biological and clinical variables that contribute to tissue mechanical heterogeneity. For example, molecular subtypes (e.g., MED12 mutations) and hormonal or therapeutic history (e.g., prior gonadotropin-releasing hormone agonist use) may theoretically modulate ECM composition, collagen cross-linking, and tissue hydration. These factors could offer a novel avenue for preoperative risk stratification and personalized surgical planning. However, given that their direct mechanical consequences in the context of fibroid morcellation remain entirely speculative, their integration into clinical practice awaits further biomechanical characterization. Ultimately, achieving the goal of overcoming “mechanical blindness” will rely not only on advancing engineering innovations but also on deepening our understanding of the unique mechanical and biological identity of each individual fibroid, thereby transitioning from standardized anatomical resection toward truly personalized, adaptive surgical interventions.

## Conclusion

The safe and efficient morcellation of uterine fibroids is fundamentally hampered by the inherent contradiction between highly heterogeneous tissue mechanics and the static design of current surgical instruments. As this review demonstrates, this mechanical mismatch—shaped by distinct growth niches and degeneration-driven remodeling—is a primary physical contributor to unpredictable fragmentation and peritoneal dissemination risks. To resolve this, a dual paradigm shift is imperative. Structurally, bio-inspired mechanisms and smart materials offer promising pathways for integrated tissue grasping and friction control. Strategically, the integration of AI-driven mechanical-imaging mapping and real-time adaptive control will offer a path toward overcoming the “mechanical blindness” of current tools. The convergence of these innovations outlines a clear roadmap for the next generation of adaptive, mechanics-aware surgical systems—transitioning fibroid surgery from a standardized anatomical procedure to a personalized intervention informed by the unique mechanical identity.
