# Supplementary material for: Overcoming Mechanical Blindness: Adaptive Surgical Instrument Design for the Heterogeneous Landscape of Uterine Fibroids
Source: Research (Wash D C). 2026 Aug 20;9:1385. doi: 10.34133/research.1385 (PMC13490745; doi:10.34133/research.1385)
Supplement: Supplementary file 1 [file research.1385.f1.pdf]

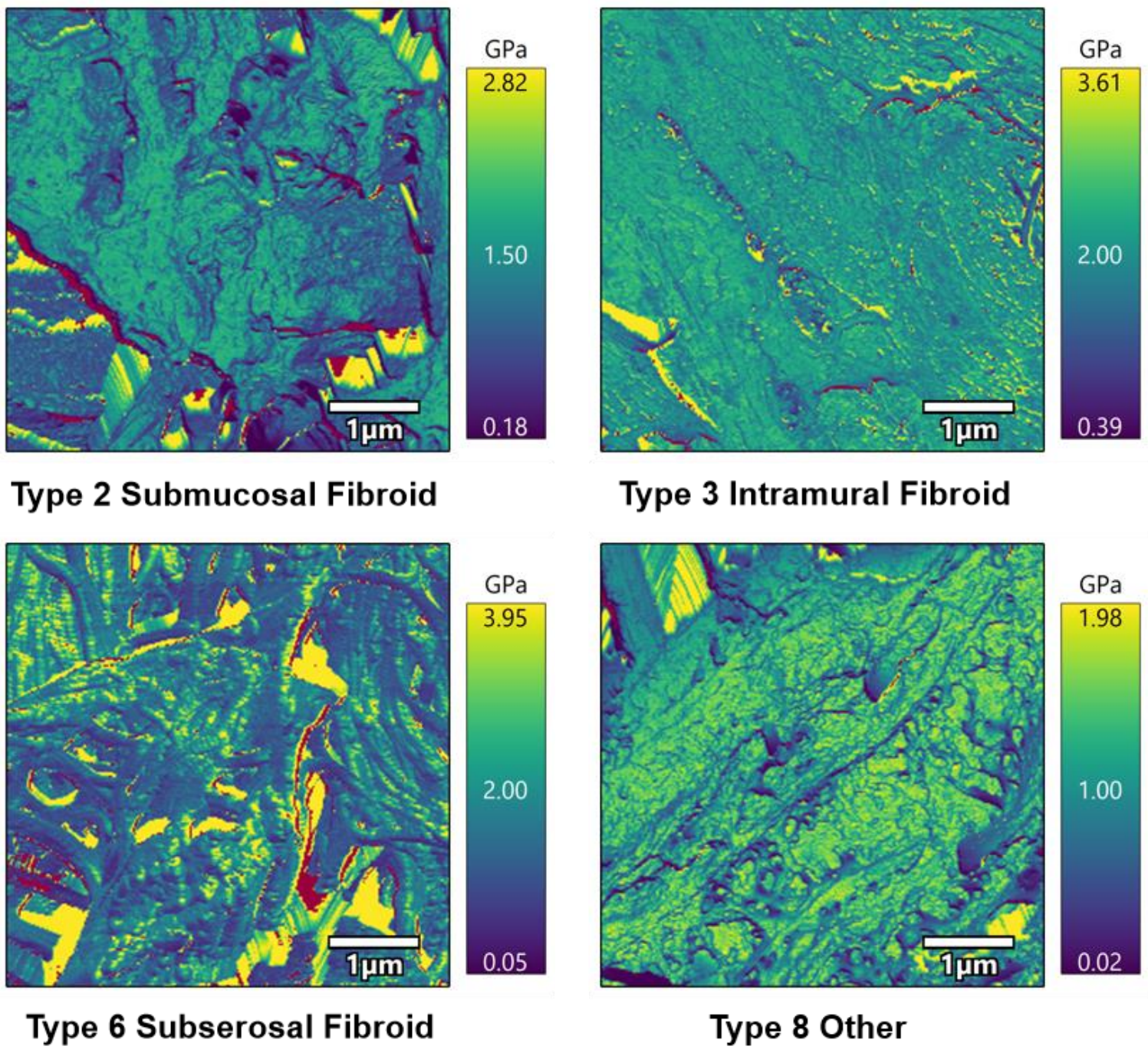

Supplementary Figure S1 : Quantitative Nanomechanical Mapping of Four Archetypal Uterine Fibroid Classifications via AFM

Spatially resolved, two-dimensional Young's modulus maps of deparaffinized histological sections (5×5\mum^{2}scan area), illustrating the structural densification gradient across distinct anatomical niches. The color scale (GPa) represents the localized static elastic modulus fitted by Sneddon's contact model. **Type 2 (Submucosal):** Displays a highly compliant, uniform matrix with minimal localized stiffening. **Type 3 (Intramural):** Exhibits an intermediate, isotropically cross-linked fibrillar network. **Type 6 (Subserosal):** Demonstrates extreme intra-sample mechanical heterogeneity, characterized by ultra-dense, zero-porosity collagenous bundles (bright yellow asperities) sharply demarcated against the stromal background, yielding the highest peak stiffness (Max). **Type 8 (Other/Cervical):** Shows a diffuse, low-gradient compliance map indicating arrested matrix compaction. Note that while the basal stroma compliance (dark blue/green regions) is fundamentally conserved across all types ($\sim 1 \sim 2$ GPa), the localized mechanical extremes (yellow/orange regions) dictate the distinct topographical contrast. Scale bar = 1 \mum for all images.

Supplementary Table S1: Quantitative Nanomechanical Properties of Four Archetypal Uterine Fibroids Derived from AFM Force Mapping

FIGO	Avg (GPa)	Max (GPa)	SHI (Max/Avg)
**Type 2 Submucosal Fibroid**	1.344	11.393	8.477
**Type 3 Intramural Fibroid**	2.099	69.803	33.256
**Type 6 Subserosal Fibroid**	2.050	82.210	40.102
**Type 8 Other**	1.094	25.036	22.885

Note: Data were extracted from high-resolution Atomic Force Microscopy (AFM) force volume mapping matrices corresponding to the representative topographical images in Figure R1. Measurements were performed on deparaffinized histological sections, with static elastic moduli quantitatively fitted via Sneddon's contact mechanics model.

- **Avg (GPa):** The average Young's modulus of the bulk extracellular matrix. The narrow conservation of this value across all archetypes ($1.094 \sim 2.099$ GPa) reflects the intrinsic baseline compliance of the dehydrated, covalently cross-linked collagenous scaffold.
- **Max (GPa):** The localized peak modulus within the mapped region, representing the absolute ceiling of spatial structural densification (zero-porosity collagen packing).
- **SHI (Stiffness Heterogeneity Index):** A dimensionless ratio calculated as Max/Avg, explicitly quantifying the intra-sample mechanical contrast. The data objectively demonstrates that while basal compliance is conserved, the localized mechanical extreme escalates topographically, culminating in the highest structural heterogeneity in Type 6 subserosal fibroids (SHI $= 40.102$).

Supplementary Table S2. Technical parameters, current status, and theoretical limitations of representative uterine fibroid morcellators

**Category**	**Representative Device (Manufacturer) [Market Status]**	**Key Technical Parameters**	**Static Limitations (Preset Deficiencies)**
**I. Motor Coring (1st Gen)**	Steiner (Karl Storz) [Superseded]	Cylindrical coring; fixed rake angle (α); 12–13 mm; 200–300 rpm; foot pedal/manual	Fixed geometry; no tissue modulus *(E)* sensing; fully manual traction prone to tissue rotation.
	X-Tract (Ethicon) [Withdrawn 2014]	15 mm; fixed rake angle; 125–1000 rpm; foot pedal/manual	No overhanging edge; manual speed preset; no real-time feedback.
	MorcDrive (WISAP) [Available regionally]	15/20 mm; serrated/wave edge; 210 rpm; manual traction; reusable	Fixed geometry; no *E* sensing; manual traction leads to slippage.
**II. Motor Peeling (2nd Gen)**	Sawalhe, Morcellex, Rotocut, VarioCarve [Mostly superseded]	Overhanging edge; 12–20 mm presets; 100–1200 rpm; foot pedal/trigger	Parameters are **pre-operative discrete presets**; no intraoperative closed-loop adaptation; no torque feedback.
	LiNA Xcise (LiNA Medical) [Available post-2014]	Cordless; 15 mm fixed; 3 mechanical positions; ~1000 rpm fixed high torque	Fixed diameter/speed; 3 positions are **discrete mechanical presets**; no *E* sensing.
	MOREsolution (BlueEndo) [Status unclear]	Peeling + fixed oscillation; 12.5–20 mm; 100–800 rpm presets	Static optimization reached its limit (van den Haak, 2016); lacks sensing & closed-loop feedback.
**III. Non-mechanical**	PKS PlasmaSOR (Olympus) [Status unclear]	Bipolar electrosurgical; 12 mm thermal probe	Non-mechanical cutting; lacks physical basis for mechanical feedback.
**IV. Wireless High-Speed (3rd Gen)**	Quanta Cyber Blade (Quanta) [Available post-2014]	Cordless; 7 speed presets (up to 6000 rpm); battery-driven	7 discrete presets; no load sensing; no closed-loop force feedback.
**V. Regional**	Wanhe Morcellator (Wanhe, China) [Available post-2014]	Rotary cutting; 10/15/18 mm; max 700 rpm	Factory preset radius *r* and speed *v*; no *E* sensing; stalling in high stiffness zones.

Category	Representative Device (Manufacturer) [Market Status]	Key Technical Parameters	Static Limitations (Preset Deficiencies)
**VI. Hysteroscopic**	TruClear / MyoSure / Resectr [Available]	Intrauterine; transcervical; manual or electromechanical	Different access route & principle; not included in adaptive laparoscopic framework.

Note:

Table Notes:

[a] Steiner (1993) superseded by Sawalhe/Rotocut; no official sales record.

[b] Ethicon (X-Tract, Morcellex, Sigma) withdrew globally in July–Aug 2014, occupying ~70% market share at that time.

[c] WISAP POWER-DRIVE MACRO remains available in Europe; 15/20 mm reusable cutting tubes, serrated/wave edge, within static paradigm.

[d] Sawalhe replaced by Rotocut; VarioCarve renamed to Morce Power Plus (Richard Wolf).

[e] LiNA Xcise (CE-marked, 2021–2024 brochures): 15 mm fixed diameter, ~1000 rpm fixed torque, 3 mechanical blade positions.

[f] Listed in Driessen et al. (2014); no peer-reviewed updates or manufacturer data post-2014.

[g] Quanta Cyber Blade: 7 discrete speed presets (max 6000 rpm), ~80 min battery life.

[h] Wanhe (China): CE & FDA approved; 10/15/18 mm diameters, max 700 rpm.

[i] Hysteroscopic devices listed for completeness; differ fundamentally from transabdominal laparoscopic morcellators.

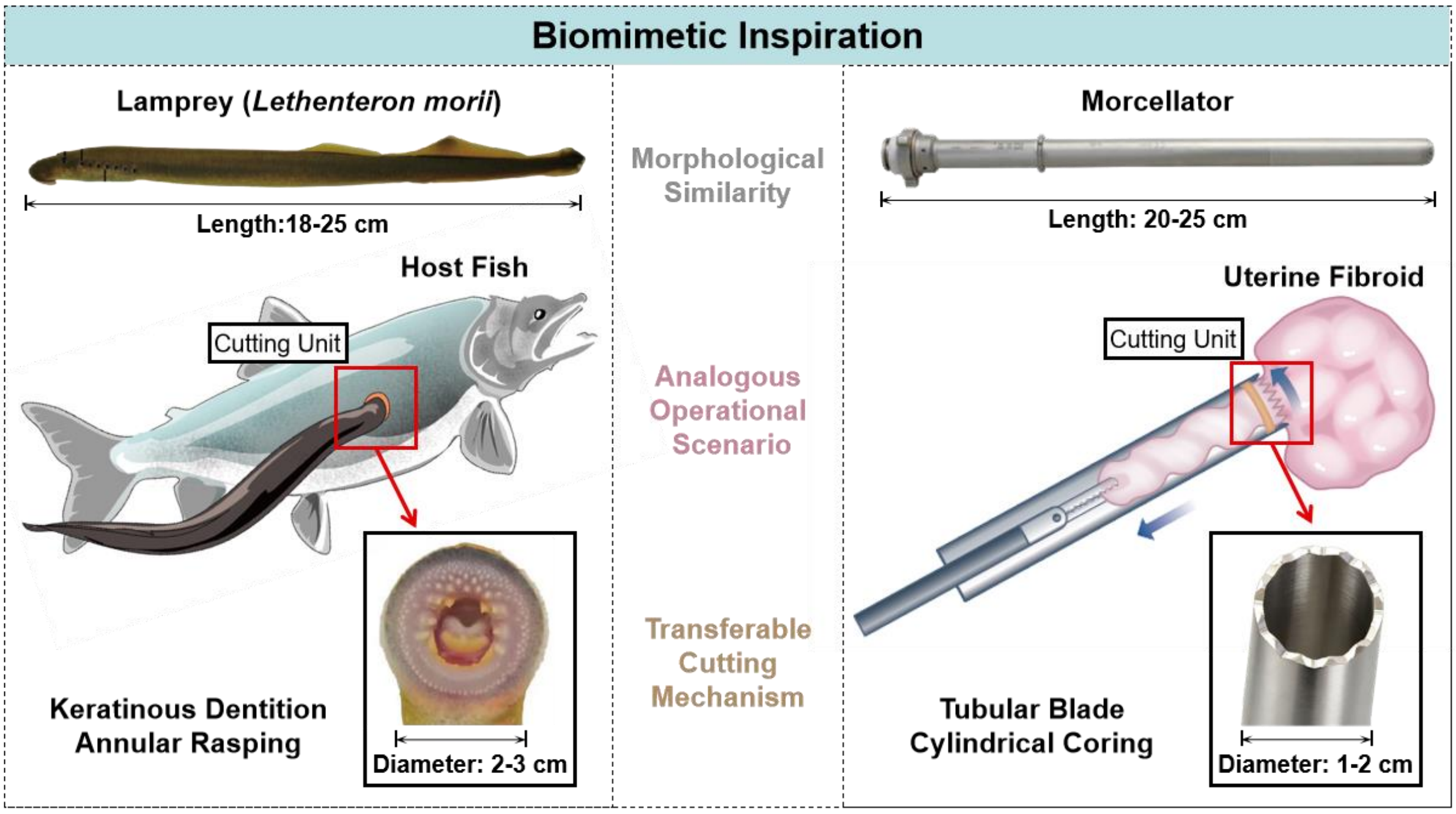

Supplementary Figure S2: Biomimetic mapping of the cutting mechanism from the Lamprey to the electromechanical uterine morcellator.

This schematic illustrates the multi-scale physical and functional analogies between the biological feeding system of the Lamprey (left) and the surgical morcellation of uterine fibroids (right). **(1) Morphological Similarity:** Both systems share a high-aspect-ratio tubular architecture (typically 18–25 cm in length) terminating in a distal cutting unit (1–2 cm in diameter). **(2) Analogous Operational Scenario:** The lamprey's biological adaptation to penetrate highly heterogeneous host tissues (e.g., hard scales versus soft flesh) directly mirrors the severe mechanical impedance mismatch encountered when morcellating heterogeneously calcified or hyalinized fibroid cores. **(3) Transferable Cutting Mechanism:** The lamprey’s keratinous dentition, which enables efficient annular rasping, serves as a direct biological blueprint for optimizing the tubular blade executing cylindrical coring. This biomimetic translation provides a tangible physical paradigm for redesigning the morcellator's cutting edge to dynamically distribute stress and mitigate abrupt high-torque loads during excision.

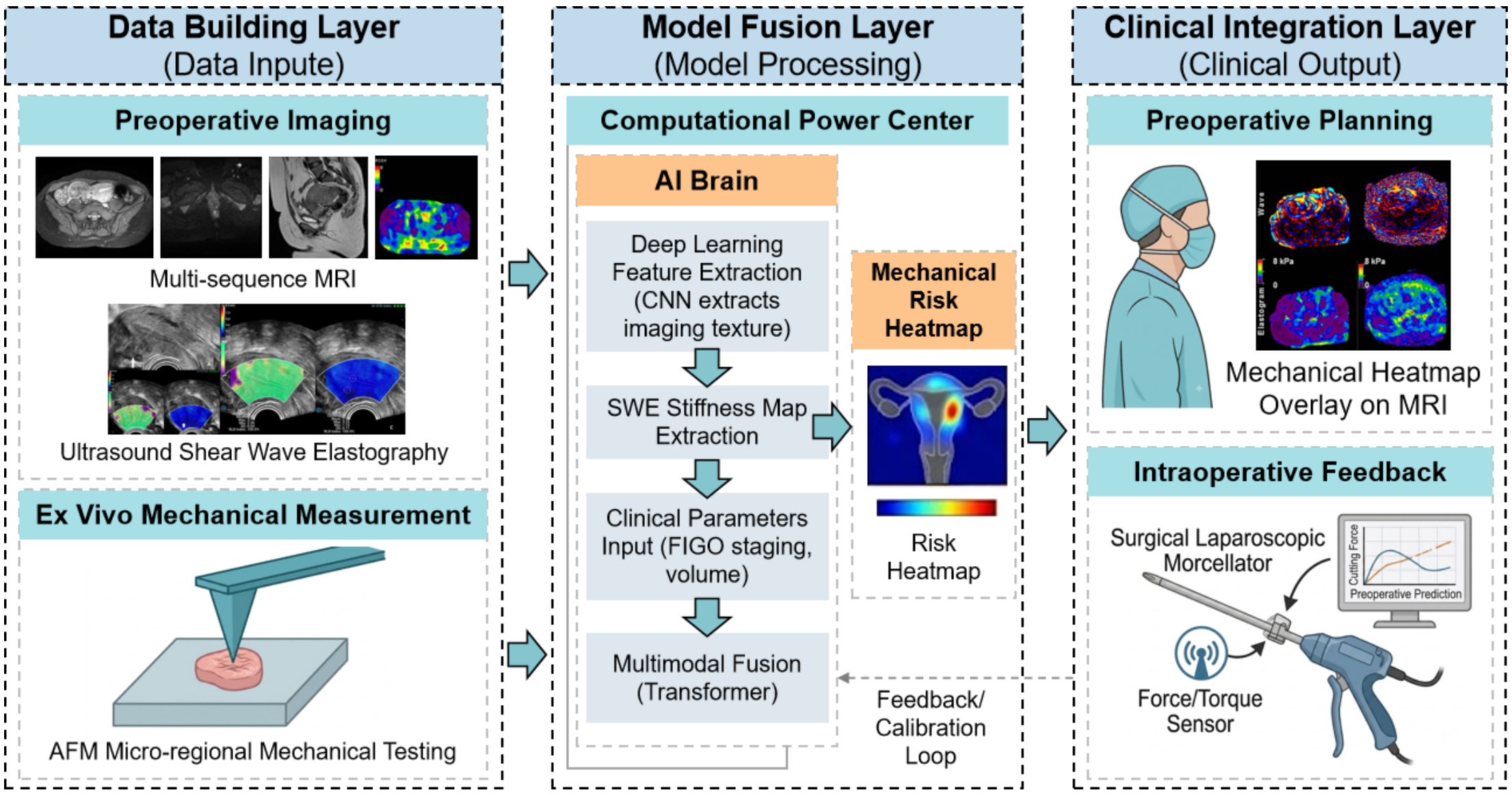

Supplementary Figure S3. Conceptual roadmap of the AI- and mechanics-informed surgical paradigm.

The framework integrates Pillars 1–2 through three layers. Left: data acquisition from preoperative multi-sequence MRI, ultrasound shear-wave elastography (SWE), and ex vivo AFM mechanical testing. Middle: multimodal AI fusion (CNN, Transformer) incorporating clinical parameters (FIGO, volume) to generate a preoperative stiffness heatmap. Right: intraoperative closed-loop feedback enabled by force/torque sensors, linking preoperative prediction with real-time adaptive cutting control.
